# Special Issue “Clinical Consequences of COVID-19”: Taking a Look at Complexity

**DOI:** 10.3390/jcm12247756

**Published:** 2023-12-18

**Authors:** Giovanni Giordano, Francesco Alessandri, Francesco Pugliese

**Affiliations:** Department of General and Specialistic Surgery, Sapienza University of Rome, Policlinico Umberto I, 00161 Rome, Italy; francesco.alessandri@uniroma1.it (F.A.); f.pugliese@uniroma1.it (F.P.)

The consequences of SARS-CoV-2 infection are far from being fully understood or accounted for. The most common and dangerous acute clinical features of the disease affect the respiratory system, and can be involved in the wide spectrum of severity at presentation; the evolution of the disease can vary enormously, ranging from asymptomatic or mild disease to critical illness with multi-organ failure and high mortality rates [1,2]. In addition to this, and soon after the outbreak of the pandemic, it became clear that several other organs were involved in the multiform clinical presentation of COVID-19, including neurological, ear, nose, and throat, gastrointestinal, ophthalmic, dermatological, cardiac, and rheumatologic manifestations. The persistence of mild symptoms and disorders for up to several months after testing negative has been designated a new nosological identity called “long covid” or “post-acute COVID-19 syndrome” [3].

In this Special Issue we collected 17 high-quality and innovative papers investigating several factors that have contributed to the clinical complexity of COVID-19: from host–pathogen interactions to different clinical manifestations, including the impact on healthcare systems and post-COVID-19 consequences.

Differences in the clinical manifestation of the disease might be influenced by intricate interactions between the virus and the patient. For example, mutations in specific SARS-CoV-2 proteins are reported to potentially influence the clinical course of the disease [4]. Additionally, demographic characteristics and lifestyle might have an impact on the severity of COVID-19 [5].

The peculiarity of patient–virus interaction may further influence the clinical management of COVID-19. In the pre-pandemic era, PCT represented a useful laboratory tool for the diagnosis and management of bacterial infections [6]. However, the study by Ceccarelli and colleagues showed that, in critically ill COVID-19 patients, abnormal procalcitonin levels do not necessarily indicate the presence of a bacterial superinfection [7]. This is particularly relevant when dealing with highly complicated patients, who are potentially susceptible to deterioration and in which a rapid differential diagnosis is crucial.

The pandemic had a significant impact on healthcare systems worldwide, and alongside COVID-19 patients, non-COVID patients also suffered different kinds of consequences [8]. For instance, the rapid spread and the severity of infection raised concerns over the safety of using immunosuppressive drugs to treat immune-mediated inflammatory diseases [9]. In addition, medical departments that were not directly managing COVID-19 patients have also experienced the consequences of the COVID-19 pandemic [10]. In fact, the need to expand bed capacity in order to accept the (un)expected, and outstanding, number of patients, led to unprecedented intra-hospital organizational efforts at the expense of reducing resources in departments that had not been converted to COVID-19 wards. Other solutions have been proposed: in order to reduce the number of beds being used at any given time, some authors explored whether early hospital discharge would have been safe or useful [11]. Last but not least, the impact of the pandemic on healthcare personnel was overwhelming [12].

Moreover, several articles published in this Special Issue focus on the post disease and long-term effects of COVID-19, including the Quality of Life of hospitalized or non-hospitalized patients, and also the physical, psychological, and social effects of the pandemic.

As Guest Editors we want to thank all of the authors, the reviewers, and the Journal of Clinical Medicine team for their precious work and support.

## Abbreviations

SAR-CoV-2	severe acute respiratory syndrome coronavirus-2
COVID-19	coronavirus disease 2019
PCT	procalcitonin
